# Critical Size Bone Defect Healing Using Collagen–Calcium Phosphate Bone Graft Materials

**DOI:** 10.1371/journal.pone.0168883

**Published:** 2017-01-03

**Authors:** William Robert Walsh, Rema A. Oliver, Chris Christou, Vedran Lovric, Emma Rose Walsh, Gustavo R. Prado, Thomas Haider

**Affiliations:** 1 Surgical & Orthopaedic Research Laboratories, Prince of Wales Clinical School, UNSW Australia, Prince of Wales Hospital, Sydney, NSW, Australia; 2 Haider Biologics, San Diego, California, United States of America; University of Notre Dame, UNITED STATES

## Abstract

The need for bone graft materials to fill bony voids or gaps that are not related to the intrinsic stability of the bone that arise due to trauma, tumors or osteolysis remains a clinically relevant and significant issue. The in vivo response of collagen–tricalcium phosphate bone graft substitutes was evaluated in a critical size cancellous defect model in skeletally mature rabbits. While the materials were chemically virtually identical, new bone formation, implant resorption and local in vivo responses were significantly different. Differences in the in vivo response may be due, in part, collagen source and processing which influences resorption profiles. Continued improvements in processing and manufacturing techniques of collagen—tricalcium phosphate bone graft substitutes can result in osteoconductive materials that support healing of critical size bone defects even in challenging pre-clinical models.

## Introduction

The need for bone graft materials to fill bony voids or gaps that are not related to the intrinsic stability of the bone that arise due to trauma, tumors or osteolysis remains a clinically relevant and significant issue. Whilst autograft can be used, the well-recognized limitations including donor site morbidity [1–5], amount and quality of autograft [6] continues to challenge researchers to develop new materials. Alternative materials to autograft from a biomaterials point of view includes a wide range bone graft substitutes [7–10] including calcium phosphates (CaP) [11–17], BioGlass [18–21] or collagen–calcium phosphate composite materials [22–25] as well as processed allografts [26] each with their own positive and negative attributes from a surgical and biological points of view. Osteoconductive materials need to be designed and manufactured that facilitates surgical handling and participates in concert at the healing site within clinically acceptable time frame. Characterizing these materials from a chemical, structural, surgical handling, radiographic and in-vivo response provides a full spectrum for understanding prior to clinical use for surgeons to make informed choices for their patients.

Pre-clinical animal studies are often a component of the Food and Drug Administration (FDA) regulatory approval process where new materials are compared to ones that are already clinically approved, so called predicates, in the 510k Submission process. Critical defect models evaluate the in vivo performance of a biomaterial in terms of new bone formation, remodeling, implant resorption and local biological effects based on radiographic and histological endpoints. The critical size bone defect has been defined as the size of an osseous defect that does not heal spontaneously with bone during the lifetime of the animal [27,28]. A variety of anatomic sites including the cranium [27–29], long bones (ulna, radius, tibia, femur) [30], cortical windows in the tibia [31–33] and confined cancellous defects on the tibia and femur [34–42] have been reported. A critical bone defect model allows a robust evaluation of the in vivo response of a material and if it can achieve the clinical goal of healing a defect that would not otherwise heal spontaneously.

While collagen based materials have a long clinical history in a number of medical devices [43,44] hypersensitivity reactions and immunological responses remains a recognized limitation [45,46]. The processing steps involved in producing any collagen based medical device can have a significant influence on the in vivo response in terms of tissue infiltration and local inflammatory response and degradation of the collagen material [44] [47–49].

This study examined the in vivo performance of two FDA approved collagen-calcium phosphate bone graft materials in a critical sized cancellous defect in skeletally mature New Zealand White Rabbits versus time. Sorrento Bone Graft Substitute (Haider Biologics LLC, San Diego, CA, USA) is a resorbable bone void filler made from a matrix of highly purified bovine tendon collagen (type I) that has high porosity beta tricalcium phosphate (TCP) granules dispersed throughout. The tendons are twice processed to remove impurities by proprietary collagen precipitation techniques and cross linked using the dehydrothermal method. The strips are made from 95% W/W Beta-TCP and 5% W/W collagen. The Beta-TCP is 95% pure TCP with up to 5% HA. Particle sizes are 500–1700 microns. Vitoss Foam Strip Bone Graft Substitute (Orthovita, Malvern, PA, USA) is a resorbable bone void filler made from a matrix of highly purified bovine skin collagen (type I) with a porous tricalcium phosphate inorganic phase. The null hypothesis of this study was that the in vivo performance in terms of new bone formation and reaction to the materials was the same. We questioned whether the differences in the quality of the collagen and type of calcium phosphate used in each material would result in a different in vivo response that could be delineated with a pre-clinical animal model.

## Methods

This study examined two collagen–tricalcium phosphate bone graft materials (Sorrento Bone Graft Substitute and Vitoss Foam Strip (Table 1) from a material science point of view and using a pre-clinical model. Five samples of each material in sterile packing were examined prior to implantation for radiographic appearance, scanning electron microscopy (SEM), Fourier Transform Infrared Spectroscopy (FTIR) and X-ray diffraction (XRD) to provide a baseline comparison. Faxitron high-resolution radiographs were taken using a Faxitron (FAXITRON BIOPTICS, LLC, Arizona, USA) and digital plates (AGFA CR MD4.0 Cassette, AGFA, Germany) (settings 24kV for 45 seconds). An AGFA Digital Developer and workstation was used to process the digital images (AGFA CR 75.0 Digitiser Musica, AGFA, Germany). The DICOM data was converted to Bitmap images using DICOM Works (ezDICOM medical viewer, copyright (c) 2002). The mean grey scale level in six regions of interest (1 cm x 2 cm) along the graft materials as well as for the entire material was examined using image J (Image J 1.47v, http://imagej.nih.gov/ij) to assess the radio-opacity and distribution of the mineral phase in the collagen sponge. The region of interest grey scale values for each material at the six sites were evaluated with a 1 way ANOVA while the overall grey scale values between the materials were evaluated with a Student’s T-Test.

**Table 1:** Allocation of defect sites and processing for this study.

		Sites at each time point			
	Description	Day 0	3 weeks	6 weeks	12 weeks
Group 1	Sorrento Bone Graft Substitute	2	7*	7*	6
Group 2	Vitoss Foam Strip	2	7*	7*	6
Group 3	Autograft (Positive control)	-	2^	2^	2
Group 4	Empty defects (Negative control)	-	-	-	2

Two sites were allocated for Groups 1 and 2 at time 0 to provide baseline for radiography and histomorphometry studies. A total of 7 sites (7*) were examined at 3 and 6 weeks for Groups 1 and 2 and distributed between Paraffin Histology (3 sites for Paraffin Histology and 4 sites for PMMA Histology)

Micro computed tomography (μCT) was performed on the same five samples using an Inveon in-vivo microcomputer tomography scanner (Siemens Medical, PA, USA). A volume of interest (13mm diameter circle by 2.7mm in depth) within the sample was chosen at random within all specimens to examine the calcium phosphate particle volume within the collagen sponge. A standard threshold level was assigned with appropriate contrast for each material to differentiate the calcium phosphate component. The calcium phosphate component volume was determined using the Siemens software for each material and differences evaluated with a Student’s T-Test.

### Stereo-zoom and environmental scanning electron microscopy (eSEM)

The macroscopic appearance of the surface directly out of the sterile packaging was examined using an Olympus stereo-zoom microscope on the same five samples. Environmental scanning electron microscopy (eSEM) was performed using a Hitachi TM 1000 Environmental electron microscope (Hitachi High-Technologies, Tokyo, Japan). The samples were mounted on stainless steel stubs with double sided carbon tape and examined at 50, 100, 250, 500, 1000 and 2000 times magnification for morphology of the CaP particles, collagen sponge, and the distribution of the CaP within the collagen network. The top, bottom and internal surfaces of the five materials of both bone graft materials were scanned at three randomly sites.

### FTIR

FTIR spectra of the inorganic (calcium phosphate) and organic (collagen matrix) phases of the materials in triplicate were obtained using a PerkinElmer Spectrum Two FT-IR spectrometer (PerkinElmer, Massachusetts, United States). The calcium phosphate was manually isolated from the collagen matrix using a fine probe. Hydroxyapatite (Product # 04238, Sigma Aldrich, Castle Hill, NSW Australia) and beta tricalcium phosphate (Cambio Ceramics, Leiden, The Netherlands) were run as a control. A type I collagen matrix (Helistat Absorbable Collagen Sponge, Integra Life Sciences, Plainsboro, NJ, USA) was used as a control for the organic phase. Spectra were acquired in transmittance mode, between the range of 4000 and 400 cm-1, averaging four scans.

### X-ray diffraction (XRD)

XRD of each the calcium phosphate materials was performed in triplicate using a PANalytical X’pert PRO Multi-purpose X-ray Diffraction System (PANalytical, Almelo, Netherlands) for the isolated CaP granules of the Sorrento and Vitoss materials as well as the HA and TCP controls. The diffraction data was acquired by exposing the powder samples to Cu-K-alpha X-ray radiation. X-rays were generated from a Cu anode supplied with 45 kV and a current of 45 mA. Phase identification was performed over the range of 10–90 degrees in 2-theta with a step size of 0.026 degrees 2-theta and nominal time per step of 148.92 s. Fixed anti-scatter and divergence slits of 0.5 degrees were used together with a beam mask of 10mm and all scans were carried out in ‘continuous’ mode. Phase identification was carried out by means of the X'Pert accompanying software program PANalytical High Score (PANalytical, Almelo, Netherlands).

### In vivo evaluation

Animal experiments were conducted following ethical clearance by UNSW Australia Animal Care and Ethics Committee (ACEC Approval # 14/116A). Twenty skeletally mature female New Zealand White rabbits were used (3.8 Kg ± 0.3 Kg). The in vivo performance of the materials was evaluated at 3, 6 and 12 weeks along with an empty defect (negative control) and autograft filled defect (positive control) (Table 1). The skeletal maturity of the animal was examined prior to surgery based on radiographic screening and confirmation of growth plate closure of the tibial tuberosity, fibula and distal femur. Radiographs were taken using a mobile X-Ray machine (Poskom, Model PXP 60HF, Image Metrix, Sydney, Australia) and digital plates (Agfa) in the lateral plane. The DICOM data was converted to Bitmap images using DICOM Works (ezDICOM medical viewer, copyright 2002). A closed growth plate was defined as a complete bony bridge and no evidence of radiolucency. Animals were weighed prior to surgery and weekly thereafter throughout the study.

The surgical procedure began with sedation via a mixture of midazolam (0.3–0.5mg/kg) and buprenorphine (0.03–0.05mg/kg) intramuscularly using a 26G needle. Anaesthesia was induced and maintained using isoflurane and oxygen inhalation during surgery. A range of isoflurane between 1–3% along with oxygen (2 litres per minute) was used. The animals were monitored for changes in vital signs (e.g. breathing and heart rate) during surgery as well as the response to pain to control the level of anaesthesia. Eye reflex and colour of mucous membranes was observed as well as oxygen levels monitored to ensure an appropriate level of anaesthesia during surgery.

The graft materials were hydrated with bone marrow aspirate (BMA) 1:1 by volume prior to implantation. BMA was harvested from the anteromedial aspect of the proximal tibia with a bone marrow aspirate needle (Sternobell, Ref RN 1925 Lot 1403475, Via L Cazzuoli, 14/16 S. Giacome Rocole, Mirandola, Italy). Autograft was harvested off the right iliac crest using a rongeur. Bilateral critical defects (6-mm in diameter and 10-mm deep) were created in the cancellous bone of the medial distal femur. A 1 cm skin incision was made to visualise medial collateral ligament (MCL) and identify the medial tubercle. The defects were prepared with a pneumatic drill under saline irrigation to minimize thermal damage with a 4.5 mm 3 fluted pyramid tip drill (Surgibit, Orthopedic Innovations, Collaroy, NSW Australia) to avoid skiving and create a pilot hole followed by a 6 mm drill to a depth of 10 mm. The base of the defect was squared off with a 6 mm flat end mill. The BMA hydrated materials were carefully placed into the defects and filled to the height of the cortex. Animals were given post-operative analgesia (Temgesic, 1ml subcutaneously) and returned to their holding cages to mobilize and weight-bear immediately post-operatively as tolerated.

Animals were euthanized via lethal cardiac injection and processed for radiographic and histological endpoints. The right and left femora were harvested and photographed using a digital camera. The general integrity of the skin incision was noted along with the macroscopic reaction of the underlining subcutaneous tissues. The femora were radiographed in the anteroposterior and lateral planes using a Faxitron (FAXITRON BIOPTICS, LLC, Arizona, USA) and digital plates (AGFA CR MD4.0 Cassette, AGFA, Germany) (settings 24kV for 45 seconds). An AGFA Digital Developer and work station was used to process the digital images (AGFA CR 75.0 Digitiser Musica, AGFA, Germany). The DICOM data was converted to Bitmap images using DICOM Works (ezDICOM medical viewer, copyright 2002). The Faxitron radiographs were evaluated for defect position as well as for any gross adverse bony reactions. Bone defect healing from a radiological point of view was examined in greater detail using micro computed tomography on all femora using an Inveon in-vivo microcomputer tomography scanner (Siemens Medical, PA, USA) in order to obtain high resolution images of the implantation site at day 0, 3, 6 and 12 weeks after implantations. Images were examined in the axial, sagittal and coronal planes to assess for any evidence of bone resorption or adjacent bony damage. The sites were examined in the sagittal plane for the healing in three sites along the 10 mm defect to assess resorption of the calcium phosphate phase as well as new bone formation in the implantation sites in a blinded fashion.

Following micro-CT the femora were fixed in 10% phosphate buffered formalin and processed for paraffin or polymethylmethacrylate (PMMA) histology. The paraffin histology was used to evaluate local tissue response using routine histology and immunohistochemistry at 3 and 6 weeks. The PMMA histology was used to evaluate local tissue response and histomorphometry of new bone formation and implant resorption versus time at 0, 6 and 12 weeks.

**Fig 1:**
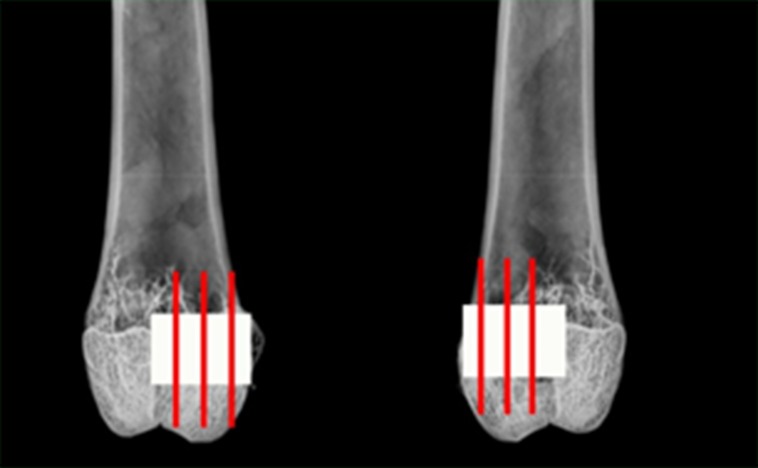
Schematic overview of where the defect was placed in the distal femur. Fig 1 provides a schematic of where the defect was placed in the distal femur as well as where the sectioning performed for micro computed tomography as well as the PMMA and Paraffin Histology.

Femurs allocated to paraffin histology were decalcified in 10% formic acid formalin and sectioned in the sagittal plane into blocks from the medial to lateral aspect of the femur in approximately 3 mm increments (Fig 1). Serial five micron sections were taken for routine histology/histopathology and immunohistochemistry. Each block was cut and stained with haematoxylin and eosin (H&E), Tetrachrome and Goldner’s Trichrome and analyzed for new bone formation and response of the graft materials as well as the local tissue reaction as per ISO 10993–6. Sections were examined using an Olympus Microscope (Olympus, Japan) with a DP72 high resolution video camera in a blinded manner.

Immunohistochemistry (IHC) for protein expression of Alkaline phosphatase (Santa Cruz Biotechnology), Cathepsin K (Santa Cruz Biotechnology, Santa Cruz, CA, USA), Matrix metalloproteinase-1 (MMP-1) (R&D Systems, Minneapolis, MA, USA), Matrix metalloproteinase-13 (MMP-13) (Oncogene Research Products, Cambridge, MA, USA), IL-6 (Abcam Plc, Cambridge, UK) and IgG (Dako Australia Pty. Ltd. Campbellfield, VIC Australia) for negative controls was performed two slides in the middle defect histology block at 3 and 6 weeks following established protocols [30,50–52]. The paraffin sections on polysine ultra plus slides were de-waxed in xylene and hydrated in a series of diluted ethanol. A pH 6.1 target retrieval citrate buffer (Dako Australia Pty. Ltd. Campbellfield, VIC Australia) was used for antigenicity recovery. Endogenous peroxidase was quenched by 0.3% hydrogen peroxide in 50% methanol for 10 minutes. Primary mouse monoclonal antibodies were incubated with the sections (one antibody per section) over night at 4°C in humidity chambers. Non-immunised mouse IgG (Dako Australia) was applied in the same manner to serve as a negative control. The final concentrations for the primary antibody and the control were determined after performing a series of optimisations of the antibodies using various concentrations. After three washes with phosphate-buffered saline with 0.05% tween-20 (PBS-T), the sections were incubated with DakoCytomation EnVision+ System-HRP labelled polymer (conjugated with anti-mouse secondary antibody) for one hour at room temperature. After four times washes with PBS-T, a Dako liquid diaminobenzidine (DAB) substrate-chromogen system was applied to the sections so that a brown end product developed at the site of the target antigens. All sections were counterstained with Harris haematoxylin and mounted with EUKITT medium (Kindler GmbH & Co., Freiburg, Germany) and cover slipped. IHC was assessed with a semi-quantitative grading scale to evaluate expression localisation and distribution in a blinded fashion in 4 high powered fields per factor per slide in a blinded manner. The grading was based on the following criteria: (-) no staining, (+) weak-staining up to 50%, (++) moderate staining 50–80% and (+++) strong staining 80–100%. Grading was performed for each group in the centre of the defect and at the host bone margin in a blinded fashion.

The femurs allocated for PMMA histology at 6 and 12 weeks were dehydrated in increasing concentrations of ethanol (70, 80, 90, 95 and 100%) prior to infiltration with methylmethacrylate (MMA) and final polymerization to PMMA. PMMA blocks were sectioned in the sagittal plane perpendicular to the long axis of the defect using a Lecia SP1600 Microtome. Three sections (~20 microns) were cut perpendicular to the defect from each site and stained with methylene blue–basic fuchsin [53] at three levels in a similar fashion to the μCT evaluation and sectioning for paraffin histology.

Two cancellous defects filled with the materials and two intact distal femurs were processed at time zero from animals from other studies to provide a baseline for the histomorphometry and micro computed tomography. Low magnification PMMA histology images (1.25x objective) of the entire defect were used to determine the amount of new bone, remaining material, void and other soft tissue in each defect based on 3 histological slides sectioned as outlined in Fig 1 using image analysis based on SORL Histomorphometry program written in MatLab (MatLab, Natick, MA, USA). The images were imported into MatLab and a region of interest defined based on the defect was identified with a polygonal tool [34]. The bone, material, marrow and void present in the defect were calculated based on colour and histological appearance. Histomorphometry data was analysed using a two way general linear model of analysis of variance followed by a Games Howell post hoc test when appropriate using SPSS for Windows.

## Results

**Fig 2:**
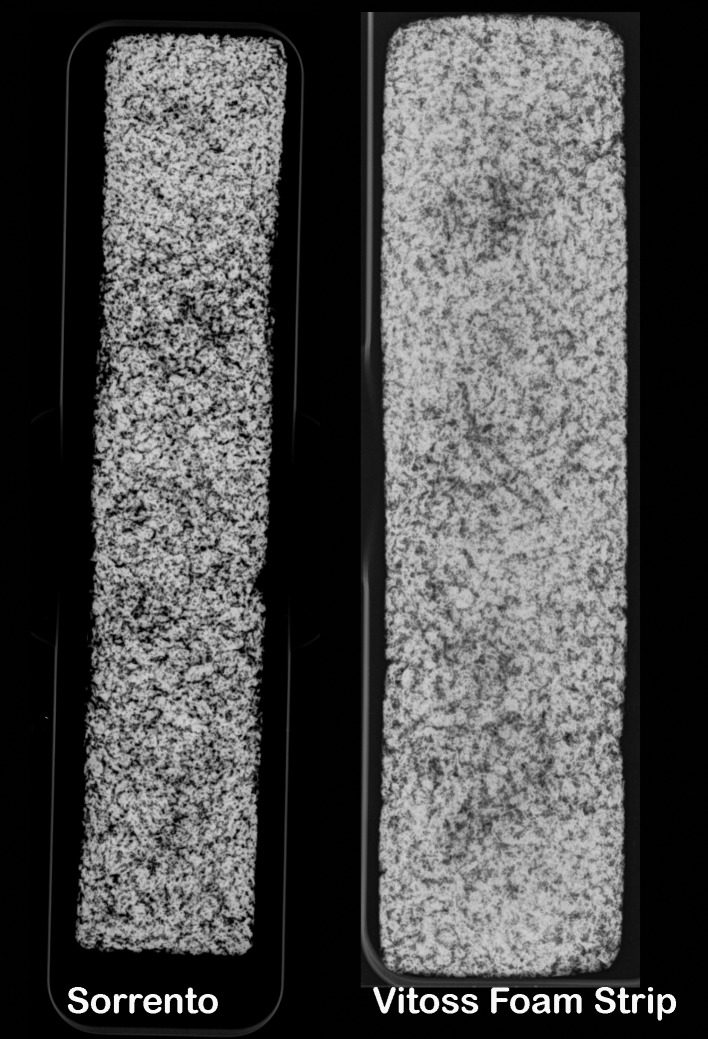
Faxitron radiographs of the materials. Fig 2 presents Faxitron radiographs of the materials as received in sterile packaging. Faxitron radiographs revealed the calcium phosphate mineral distributed in the material within both the materials. Both graft materials had a uniform radio-opacity along the 10 cm length in all five sample examined. No differences were detected within graft materials for grey scale for the 6 region of interest (P>0.05). The overall grey scale value for Sorrento Bone Graft Substitute was however great than that compared to Vitoss Foam Strip (P<0.05).

Both graft materials had a uniform radio-opacity along the 10 cm length in all five sample examined (Fig 2). No differences were detected within graft materials for grey scale for the 6 region of interest (P>0.05). The overall grey scale value for Sorrento Bone Graft Substitute was however great than that compared to Vitoss Foam Strip (P<0.05). Similarly, the micro-computed tomography analysis revealed a great CaP component volume for the Sorrento compared to the Vitoss Foam Strip (P<0.05).

**Fig 3:**
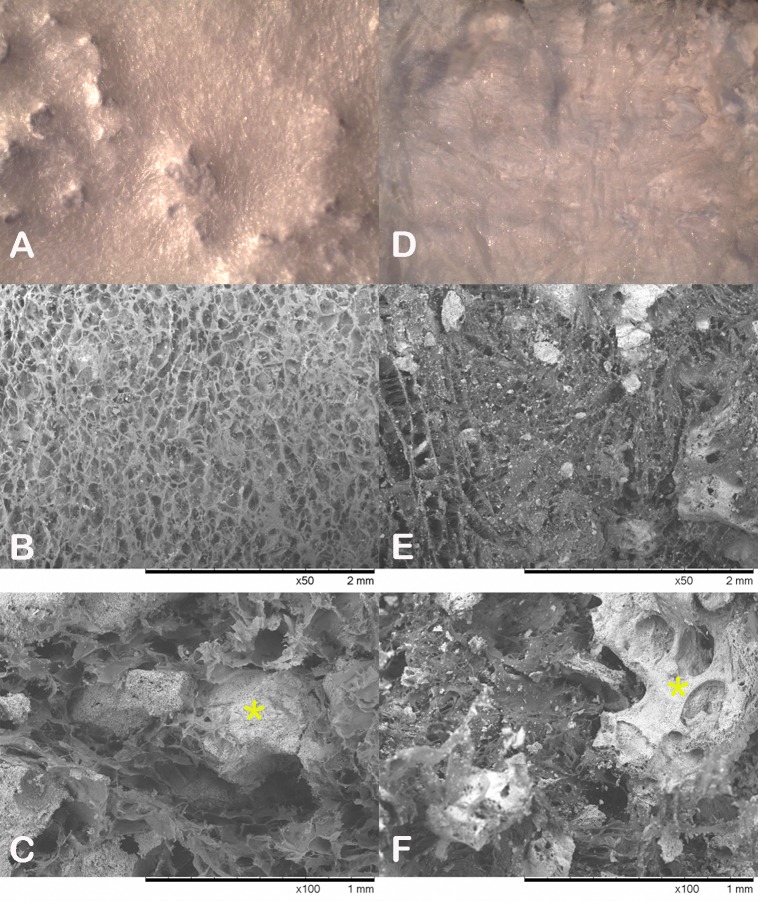
Stereozoom and electron microscopy of the materials. Fig 3 presents stereo-zoom and electron microscopy images revealing qualitative differences in the surface and interior of the graft materials. The Sorrento Bone Graft Substitute (A, B, C) and Vitoss Foam Strip (D,E, F) had a different surface structure based on stereozoom images (A vs D) as well as environmental scanning electron microscope image of the surface (B vs E) and the interior (C vs F).

Stereo-zoom and electron microscopy revealed qualitative differences in the surface and interior of the graft materials (Fig 3). The type I collagen component of each graft material appeared white and the calcium phosphate components could be observed to be covered by the collagen. The surface of the Sorrento graft material appears to be more uniform compared to the Vitoss foams Strip. Environmental scanning electron microscopy images are presented a number of interesting features between the two graft materials (Fig 3). Differences were noted for both materials when comparing the surface and in the middle of the sample. The CaP component in the Sorrento material appeared to be covered by the collagen matrix while some CaP granules were present on the surfaces of the Vitoss Foam Strip. Sorrento material presented a uniform open collagen pore structure (100–150 micron) along the surface which was impregnated with CaP granules when viewed in cross section (Fig 3). The collagen matrix appeared evenly distributed with no clumping or disorganized collagen structure. The CaP granules were intimate with the collagen matrix but the surface of these granules remained free of a collagen covering and demonstrated microscale roughness. The collagen matrix morphology in the Vitoss Foam Strip appeared denser and less open pores. The CaP component of the Vitoss Foam Strip revealed a similar roughened appearance by the presence of micro-porosity not seen with the Sorrento material. The collagen matrix also appeared to be more fibril in nature for the Vitoss foam strip and cover the CaP phase along with the presence of smaller granules of CaP (Fig 3).

**Fig 4:**
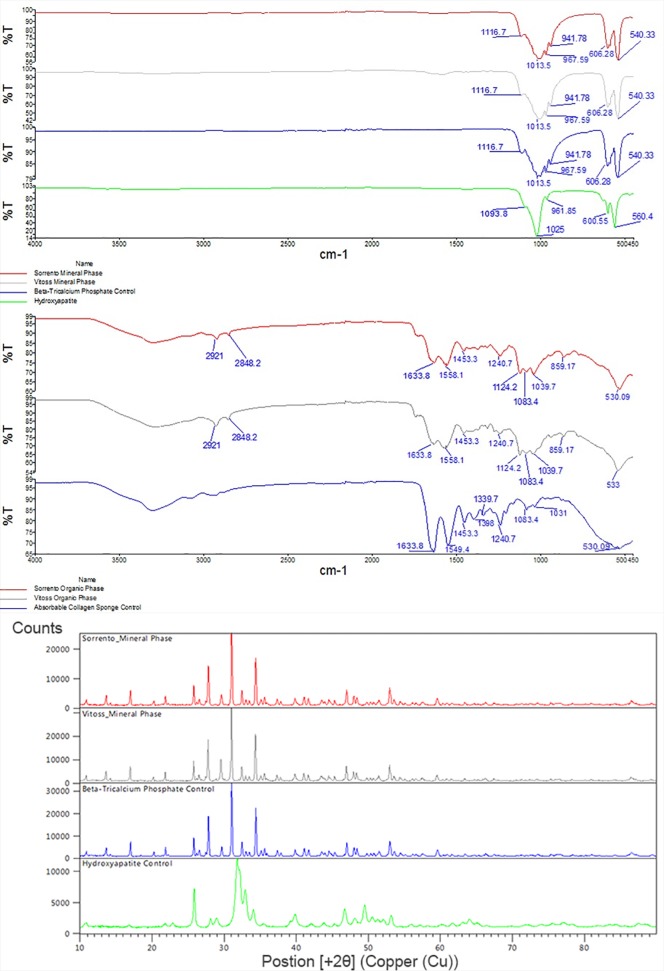
Fourier Transform Infrared Spectroscopy and X-ray Diffraction. Fig 4a The FTIR spectra for the calcium phosphate portions of the graft are shown in the top panel. The Sorrento Bone Graft Substitute (red) and Vitoss Foam Strip (Grey) compared well to each other were both similar to the tricalcium phosphate control (Blue) and differed compared to the hydroxyapatite control (green) [55]. The FTIR spectra for the organic portions of the graft are shown in the bottom panel. The Sorrento Bone Graft Substitute (red) and Vitoss Foam Strip (Grey) compared well to each other were similar to the Collagen control (Blue). The typical amide peaks of collagen were present in all samples [54]. There was however evidence of the calcium phosphate residual material as evident by the phosphate peaks at approximately 1100–950 cm-1. Fig 4b The XRD diffraction patterns for the calcium phosphate portions of the graft are shown in the top panel. The Sorrento Bone Graft Substitute (red) and Vitoss Foam Strip (Grey) compared well to each other were both similar to the tricalcium phosphate control (Blue) and differed compared to the hydroxyapatite control (green) [55].

In contrast to the morphological evaluations reported above, the FTIR spectra (Fig 4A) and X-ray diffraction (XRD) patterns (Fig 4B) revealed very similar chemical signatures for calcium phosphate portions of the bone graft materials. The chemical signature demonstrated the CaP phases to be consistent with tricalcium phosphate based on FTIR and XRD data as reported in the literature [54,55]. Further analysis of the collagen portion of the material revealed similar FTIR spectra with the typical amide peaks of collagen present [54]. There was however evidence of some calcium phosphate residual material as evident by the phosphate peaks at approximately 1100–950 cm-1 that was not removed upon manual separation of the materials.

Surgical handling revealed both materials to readily hydrate with BMA which improved handling. The defects were easily filled with the graft materials. All animals recovered uneventfully and were healthy throughout the study. All wounds healed without complication and no adverse reactions were noted during harvest of the femurs.

**Fig 5:**
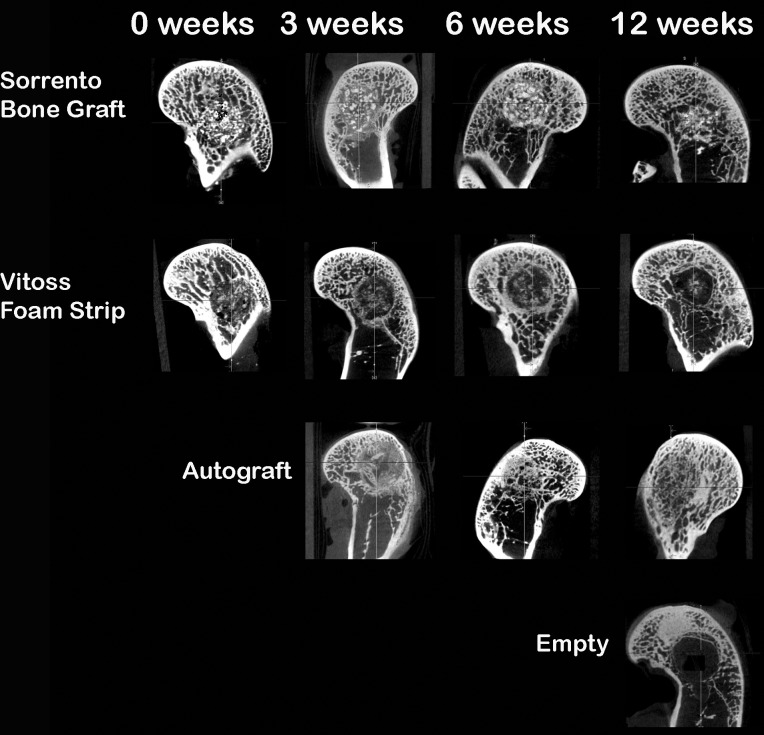
Micro-computed tomography versus time. Fig 5 Micro-CT provided a unique insight into the radiographic signal and performance of the two materials and healing of the defect. Increased signal intensity suggesting newly formed woven bone was seen at the defect margins as well as extending into the centre of the defects with the Sorrento BMA material at 3 weeks that progressed with time. In contrast, micro-CT for the Vitoss Foam Strip BMA group did not demonstrate new bone formation at 3 weeks while some new bone formation at the margins could be seen at 6 weeks this did not progress into the centre of the defect with time. The autograft treated defects healed well with time while the empty defects remained empty demonstrating the critical nature of the model. These findings agreed well with the histology results for all defects.

Anteroposterior (AP) and lateral radiographs confirmed the confined cancellous nature of the defect in the medal distal femur and did not reveal any adverse bony reactions for either material at any time. The Faxitron radiographs were of little to no value in evaluating bone healing or implant resorption and are not presented micro-computed tomography, on the other hand, provided a unique insight into the radiographic signal and performance of the two materials. Increased signal intensity suggesting newly formed woven bone was seen at the defect margins as well as extending into the centre of the defects with the Sorrento BMA material at 3 weeks which progressed with time (Fig 5). Intimate bone–CaP contact as well as bone remodelling and CaP resorption was noted with time. In contrast, micro-computed tomography for the Vitoss Foam Strip BMA group did not demonstrate new bone formation at 3 weeks while some new bone formation at the margins could be seen at 6 weeks this did not progress into the centre of the defect with time. The autograft treated defects healed well with time while the empty defects remained empty demonstrating the critical nature of the model (Fig 5).

**Fig 6:**
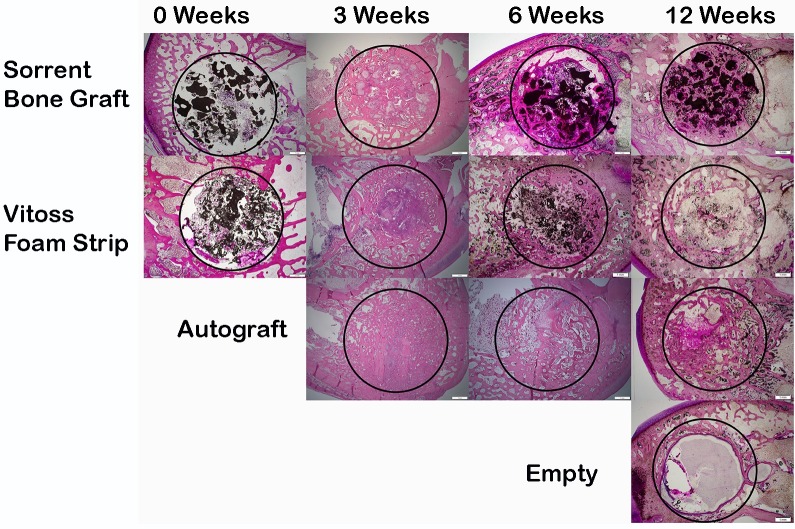
Histology Fig 6 Histology confirmed the micro computed tomography findings in terms of new bone formation and implant resorption versus time. The histology images were taken with a 1.25x objective to provide an overview of the site with the defect outlined with a black circle. PMMA histology is presented for time 0 and the 6 and 12 week time points for Sorrento and Vitoss Foam Strip and Empty defects at 12 weeks. These sections were stained with methylene blue/basic fuschin. The 3 week data is from paraffin histology stained with H&E for all groups. The 6 week histology for autograft is also from paraffin histology stained with H&E. The Sorrento BMA material supported new bone formation as early as 3 weeks with resorption of the mineral phase with time. In contrast, Vitoss Foam Strip BMA group did not demonstrate significant new bone formation at 3, 6 or 12 weeks in the middle of the defect although some new bone formation at the defect margins. The autograft treated defects healed well with time while the empty defects remained empty demonstrating the critical nature of the model.

**Fig 7:**
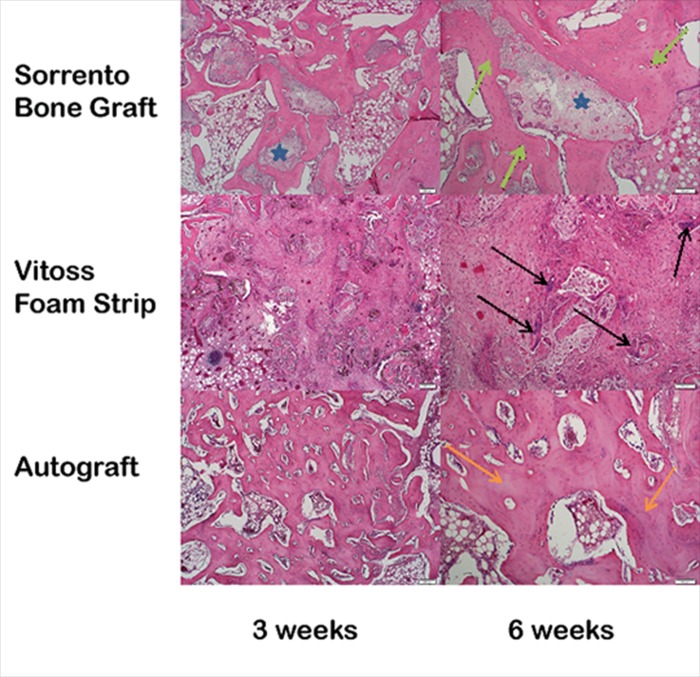
Higher magnification histology. Fig 7 Higher magnification histology in the middle of the defect is presented at 6 weeks (4x and 10x objectives and stained with H&E) to illustrate the in vivo differences observed between Sorrento Bone Graft Substitute, Vitoss Foam Strip and Autograft. The Sorrento BMA material supported new bone formation with direct bone formation on the surface of the calcium phosphate phase (star) and newly formed bone (green arrows) marrow spaces at 6 weeks. In contrast, Vitoss Foam Strip BMA group did not demonstrate significant new bone formation 6 weeks in the middle of the defect with noted evidence of inflammatory cells (black arrows) and fibrous tissue response. The autograft treated defects demonstrate new bone formation directly on the remaining graft material present (orange arrows) in the defect as well as newly formed marrow spaces.

Paraffin and PMMA histological confirmed the micro-computed tomography findings observed at 3, 6 and 12 weeks. The Sorrento BMA material outperformed the Vitoss Foam Strip BMA group at all time points in terms of newly forming bone on and through the materials, lack of fibrous tissue in the defect and normal bone remodeling. New bone formation throughout the defect was observed at 6 weeks with remodelling and marrow spaces at 12 weeks for defects filled with Sorrento BMA as well as the autograft, positive control group. The increased signal intensity observed in the micro computed tomography with Sorrento BMA observed in Fig 5 at 3 and 6 weeks was indeed newly forming woven bone found at the host bone margins as well as deep into the defect (Figs 6 and 7). A direct comparison of the micro-computed tomography data and histology demonstrated the radio-opacity observed within the Vitoss Foam Strip BMA treated defects to be residual material and fibrous tissue rather than newly forming bone (Figs 5–7). Complete defect healing with new and remodelled bone was not achieved by 12 weeks with Vitoss Foam Strip hydrated with BMA with presence of multinucleated giant cells, lymphocytic infiltrate and fibrous tissue (Fig 7). The autograft treated defects, positive control group, performed well and paralleled the results observed with the Sorrento BMA with newly forming bone observed at 3 week which improved and healed the defect with time. Residual autograft material could be discerned at 3 and 6 weeks and appears as portions of graft material with the absence of cells (Fig 7). The defects that were left empty negative control remained empty filled with fibrous and fatty tissues at 12 weeks (Fig 6).

**Fig 8:**
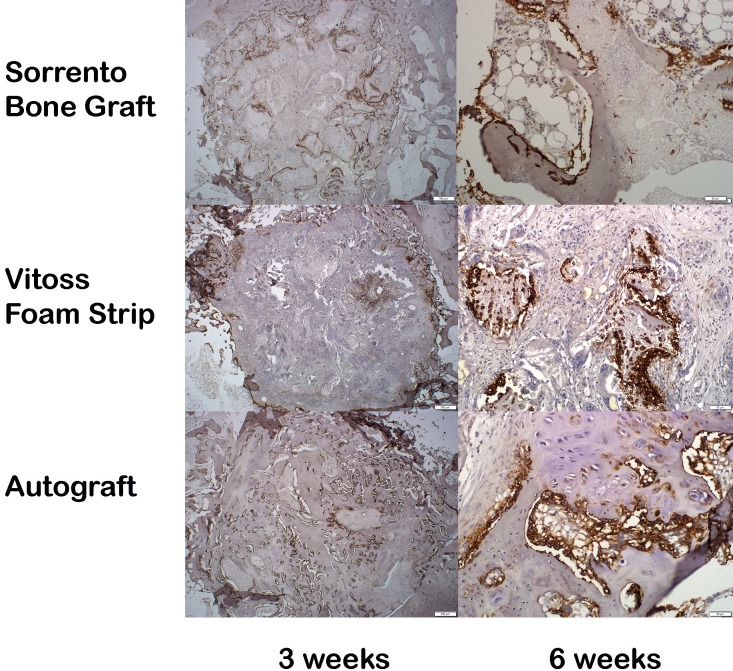
Immunohistochemistry expression. Fig 8 Immunohistochemistry results for Alkaline phosphatase, (ALP) is a widely accepted bone marker activity and osteogenic potential, is presented for Sorrento Bone Graft Substitute, Vitoss Foam Strip and Autograft at 3 weeks and 6 weeks. The 3 week panels are examined at a low magnification (1.25x objective) while the 6 week presents the results in the center of the defect (20x objective). Positive expression for ALP is noted by the brown staining. The results at 3 weeks demonstrate ALP expression in the osteoblasts throughout the defects for defect treated with Sorrento Bone Graft Substitute as well as Autograft while defects treated with Vitoss Foam Strip shows little to no expression apart from the defect margins. IHC for ALP at 6 weeks shows the remodeled bone in the Sorrento Bone Graft Substitute treated defects with continued expression in the osteoblasts lining the newly formed bone, the presence of some fibrous tissue and some ALP expression for Vitoss Foam Strip and expression of ALP by the active osteoblasts in the Autograft treated defects.

Immunohistochemistry (IHC) was performed to evaluate protein expression in the early phases of healing at 3 and 6 weeks using the paraffin sections. Table 2 represents an overall summary of the percentage of staining intensity for each antibody factor tested. Grading was performed for each group in the defect center as well as host bone margins. Consistent with the new bone formation or lack of new bone formation observed in the paraffin and PMMA, the IHC demonstrated positive expression of alkaline phosphatase at the defect margins as well as in the deeper into the defect for the Sorrento BMA and Autograft groups at 3 weeks which increased at 6 weeks, while the Vitoss BMA group lagged behind with little alkaline phosphatase expression with expression only at the defect margins at 3 weeks and some areas of increased expression at 6 weeks (Fig 8).

**Table 2:** 

		Site					
		Middle			Margins		
Factor	Time	Sorrento + BMA	Vitoss Foam Strip + BMA	Autograft	Sorrento + BMA	Vitoss Foam Strip + BMA	Autograft
Cathepsin-K (CAT-K)	3 weeks	+++	++	+	+	++	+
Alkaline phosphatase (ALP)	3 weeks	++	-	+++	++	++	+++
Matrix metalloproteinase-1 (MMP-1)	3 weeks	++	+	-	-	+	-
Matrix metalloproteinase-13 (MMP-13)	3 weeks	+++	++	++	+++	++	+++
Interleukin-6 (IL-6)	3 weeks	-	-	-	-	-	-
mIgG	3 weeks	-	-	-	-	-	-
Cathepsin-K (CAT-K)	6 weeks	+++	++	+	++	++	+
Alkaline phosphatase (ALP)	6 weeks	-	-	++	+++	++	++
Matrix metalloproteinase-1 (MMP-1)	6 weeks	++	+	-	+	-	-
Matrix metalloproteinase-13 (MMP-13)	6 weeks	+++	+++	+	+++	+++	+
Interleukin-6 (IL-6)	6 weeks	-	-	-	-	-	-
mIgG	6 weeks	-	-	-	-	-	-

Grading was performed for each group at 2 sites–middle of the defect and margin of the defect.

The grading was based on the following criteria

(-) no staining

(+) weak staining up to 50%

(++) moderate staining 50–80%

(+++) strong staining 80–100%.

CAT-K expression was noted for the osteoclastic-like cells on the surface of the calcium phosphate granules for Sorrento BMA and Vitoss BMA treated defects and to a lesser extent on residual autograft present in the defect site. Some expression was also observed in fibroblasts, osteocytes and osteoblasts lining the new bone at 3 and 6 weeks (Table 2). MMP-1 expression was present for both materials at 3 and 6 weeks and was elevated compared to autograft at both time points (Table 2). Similarly, MMP-13 expression was also found with both materials at 3 and 6 weeks while the expression for autograft appeared to decrease with time. IL-6 expression was not detected for any group at 3 or 6 weeks.

**Fig 9:**
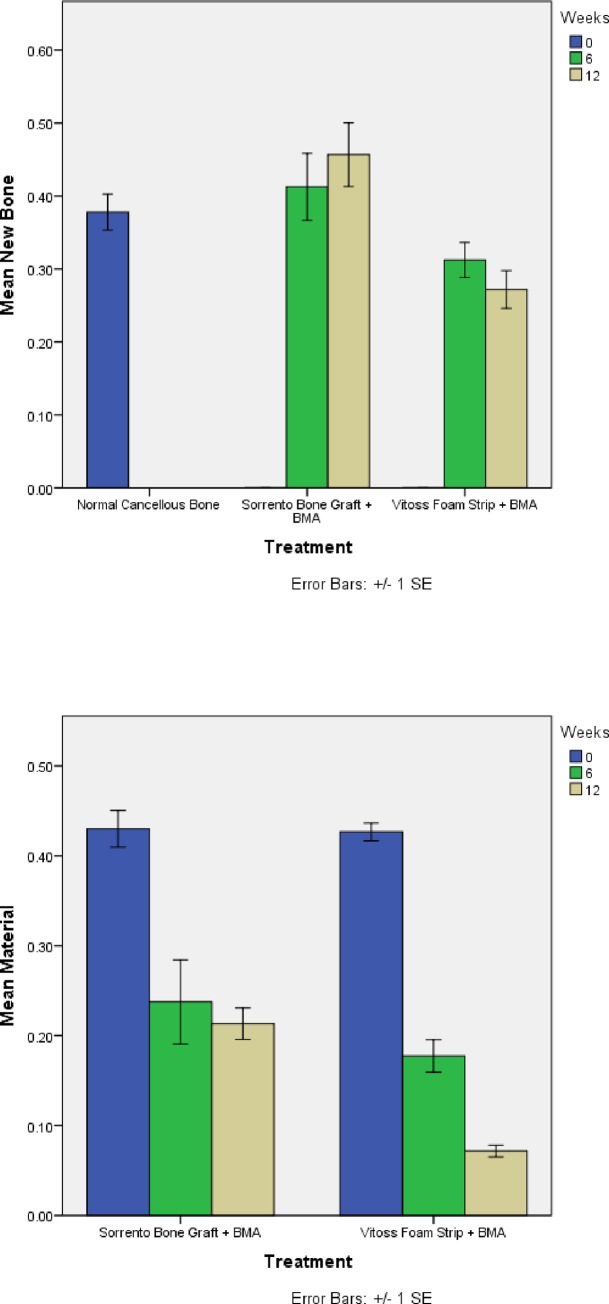
Histomorphometry. Fig 9a) Mean new bone formation histomorphometry revealed Sorrento Strip + BMA outperformed Vitoss Foam Strip + BMA (P<0.05) at 6 and 12 weeks. New bone formation increased slightly for the Sorrento Strip + BMA while the Vitoss Foam Strip + BMA decreased slightly. This was consistent with additional new bone formation in the Sorrento Strip treated sights as well as remodeling of the newly formed bone. The results of the Vitoss Foam Strip + BMA for new bone formation were consistent with remodeling of the newly formed bone and lack of additional bone formation at 12 weeks. Fig 9b) Mean material histomorphometry revealed Sorrento Strip + BMA and Vitoss Foam Strip + BMA resorbed with time from 0 to 6 and to 12 weeks after surgery.

Histomorphometry of 3 slides for each defect (Fig 1) was used to determine the amount of new bone, remaining material, void and other soft tissue. Analysis revealed significantly more new bone formation for defects treated in the Sorrento BMA compared to the Vitoss BMA treated defects at 6 and 12 weeks (P<0.05) (Fig 9A). Both materials resorbed with time compared to initial implantation (time 0) at 6 weeks with no statistical differences in graft resorption noted for the Sorrento BMA material between 6 and 12 weeks while the Vitoss BMA continued to resorb (Fig 9B). Similar, both materials had a decrease in other soft tissue with time (data not shown).

## Discussion

Bone graft substitutes provide surgeons with an “off the shelf” alternative to aid in the healing of defects or spinal fusion. The ideal material should handling well during surgery, be easily implanted into the defect and ultimately function as an osteoconductive scaffold to support and heal the defect. The number and variety of bone graft materials that have been developed as well as applied clinically is remarkable [7–10] suggesting we have yet to achieve the optimal material for clinical use. Bone graft substitutes based on the primary constituents of the unique composite, type I collagen and calcium phosphate, is a logical choice. Biomaterials based on collagen have a long history in a range of clinical applications prior to use as bone graft materials [43,44,56]. The elegance and complex nature of the type I collagen molecule has resulted in a number of challenges in terms of processing for biomaterials in terms of antigenicity and immunogenicity [45,46].

Collagen–tricalcium phosphate bone graft substitutes were examined in the current study by characterisation from a material science point of view and in vivo using a critical size cancellous defect model [20,38,40–42,57] and at the cell and tissue level using histology and immunohistochemical expression MMP-1, MMP-13 and Cathepsin K [58–60] factors known to be involved in the degradation of the components of bone. While the inorganic tricalcium phosphate and organic collagen matrix of these materials as well as the BMA hydration and surgical handling were virtually identically, bone healing, implant resorption and local tissue reactions however revealed a different result. The Sorrento bone graft material hydrated with BMA outperformed Vitoss Foam strip hydrated with BMA in the primary purpose of a bone graft a material which is to heal the defect with new bone and remodelled with time. This may be due, in part, to the collagen source (skin versus tendon), amount, quality, processing (crosslinking method, washing etc.) and the overall collagen matrix ultrastructure (observed in SEM) as well as CaP component differences (amount, porosity, size). The raw material for the Sorrento bone graft collagen component is bovine tendon while the Vitoss Foam strip collagen component is derived from skin. Subtle differences in collagen processing including crosslinking [48] as well as source material may account for the differences observed at the histological level [45,46] and defect healing. The Sorrento bone graft material has a 5% W/W collagen and 95% TCP ratio while Vitoss Foam Strip has a 20% W/W collagen and 80% TCP ratio. Differences in graft resorption appeared to influence the in vivo response in terms of new bone formation at the defect margins as well as in the middle (Fig 7) with the prolonged inflammatory response observed with Vitoss potentially due to a slower breakdown of the collagen material and continued presence of collagen breakdown products, which have been reported to be chemotactic for inflammatory cells induce a mild reaction [47,48].

The immunohistochemical data on protein expression (Table 2) provides an additional level of evidence related to the early response following implantation. In vivo expression of MMP-1, MMP-13 and Cat-K was found for both materials suggesting a similar pathway in the enzymatic breakdown of the components while the response to morselized autograft within the defect was less robust (Fig 7). Collagen degradation can be influence by many endopeptidases [49]. While MMP-1 and 13 and Cat K expression was present for all materials in this study, differences in expression in other matrix metalloproteinases (MMP-2, 3, 9 and 10) as well as other cysteine and serine proteinases that may continue influence an inflammatory response through would be an interesting area of investigation in the future.

Differences in the microstructure of the inorganic tricalcium phosphate components (porosity, particle size and firing conditions) that did not alter the FTIR spectra or XRD patterns of the materials could also account for the differences observed in vivo. The TCP component in Sorrento was a denser granule while the TCP in Vitoss Foam strip was more porous. A similar finding was reported for the osteoinductivity of two calcium phosphates by Yuan and colleagues [13] with respect to similar chemistry and different in vivo results. These authors reported that two calcium phosphate materials with similar chemical and crystallographic structures had completely different in vivo performance [13]. One CaP was inductive while the other was not. Faxitron radiographs and micro computed tomography did however reveal differences in the radio-opacity between the materials due to the difference in the size, porosity and distribution of the calcium phosphate phase of the materials. The Sorrento graft material appeared less dense compared to the Vitoss Foam Strip.

The experimental model of a critical size defect in cancellous bone of the skeletally mature NZ White Rabbit provides a robust experimental means to evaluate the in vivo response of potential bone graft substitutes [20,32,33,40–42,57]. Nevertheless, it remains limited in the pre-clinical nature. The distal femur defect model is however relatively simple to perform and well tolerated by the animals post-operatively. We find the use of a 3-fluted pyramidal tip drill to avoid skiving off the bone provides additional control in creating the defect. In terms of endpoints, radiographs provide little value when micro computed tomography is also performed. Micro computed tomography is limited to a qualitative analysis when calcium phosphate bone grafts are present due to the similar grey scale values to bone. Nevertheless, micro computed tomography is a useful tool to assess overall defect healing in multiple planes and any adverse bony reactions well beyond radiographs. Evaluating the in vivo response with decalcified and non-decalcified histology provides interesting insight into the in vivo responses of materials and should be considered in light of the micro computed tomography data as well as the location with the defect.

The current study is limited considering we did not evaluate time points prior to 3 weeks. Examining the in vivo response prior to 3 weeks may provide further differences between materials and the initial cellular reactions that dictate healing. We also did not examine the full spectrum of enzymes that can degrade collagen which may influence the inflammatory response through the healing. Further improvements in biomaterial processing and development as well as our ability to differentiate them in pre-clinical models to provide insight into bone healing is hoped to improve patient outcomes.
